# Impact of the Coronavirus Disease 2019 Pandemic on Substance Use Disorder Risk Among People Living With Human Immunodeficiency Virus (HIV) Enrolled in HIV Care in the United States: An Interrupted Time Series Analysis

**DOI:** 10.1093/ofid/ofae491

**Published:** 2024-08-26

**Authors:** Jennifer P Jain, Megan J Heise, Nadra E Lisha, Carlos H Moreira, David V Glidden, Greer A Burkholder, Heidi M Crane, Jeffrey M Jacobson, Edward R Cachay, Kenneth H Mayer, Sonia Napravnik, Richard D Moore, Carol Dawson-Rose, Mallory O Johnson, Katerina A Christopoulos, Monica Gandhi, Matthew A Spinelli

**Affiliations:** Division of Prevention Science, University of California, San Francisco, California, USA; Division of HIV, Infectious Diseases, and Global Medicine, University of California, San Francisco, California, USA; Department of Epidemiology and Biostatistics, University of California, San Francisco, California, USA; Division of HIV, Infectious Diseases, and Global Medicine, University of California, San Francisco, California, USA; Department of Epidemiology and Biostatistics, University of California, San Francisco, California, USA; Division of Infectious Diseases, University of Alabama at Birmingham, Birmingham, Alabama, USA; Division of Allergy and Infectious Diseases, University of Washington, Seattle, Washington, USA; Divsion of Infectious Diseases, Case Western Reserve University, Cleveland, Ohio, USA; Division of Infectious Diseases, University of California, San Diego, California, USA; Department of Medicine, Harvard University, Boston, Massachusetts, USA; Division of Infectious Diseases, University of North Carolina, Chapel Hill, North Carolina, USA; Division of Infectious Diseases, Johns Hopkins University, Baltimore, Maryland, USA; School of Nursing, University of California, San Francisco, California, USA; Division of Prevention Science, University of California, San Francisco, California, USA; Division of HIV, Infectious Diseases, and Global Medicine, University of California, San Francisco, California, USA; Division of HIV, Infectious Diseases, and Global Medicine, University of California, San Francisco, California, USA; Division of HIV, Infectious Diseases, and Global Medicine, University of California, San Francisco, California, USA

**Keywords:** COVID-19, depression symptoms, HIV/AIDS, LGBTQ, substance use disorder

## Abstract

**Background:**

Rising overdose deaths globally and increased social isolation during the coronavirus disease 2019 (COVID-19) pandemic may have disproportionately impacted people with human immunodeficiency virus (PWH) with substance use disorders (SUD). We examined trends in SUD risk among PWH before and after the COVID-19 shelter-in-place (SIP) mandate.

**Methods:**

Data were collected between 2018 and 2022 among PWH enrolled across 8 US sites in the Centers for AIDS Research Network of Integrated Clinical Systems cohort. We evaluated changes in moderate/high SUD risk after SIP using interrupted time series analyses.

**Results:**

There were 7126 participants, including 21 741 SUD assessments. The median age was 51 (interquartile range, 39–58) years; 12% identified as Hispanic or Latino/Latina, 46% Black/African American, and 46% White. Moderate/high SUD risk increased continuously after the pandemic's onset, with 43% (95% confidence interval [CI], 40%–46%) endorsing moderate/high SUD risk post-SIP, compared to 24% (95% CI, 22%–26%) pre-SIP (*P* < .001). There were increases in the use of heroin, methamphetamine, and fentanyl, and decreases in prescription opioids and sedatives post-SIP. Further, there was a decrease in reported substance use treatment post-SIP compared to pre-SIP (*P* = .025).

**Conclusions:**

The rising prevalence of SUD through late 2022 could be related to an increase in isolation and reduced access to substance use and HIV treatment caused by disruptions due to COVID-19. A renewed investment in integrated substance use treatment is vital to address the combined epidemics of substance use and HIV following the COVID-19 pandemic and to support resilience in the face of future disruptions.

## Teaser

At-risk substance use among people with HIV (PWH) increased over 2 years following COVID-19 shelter-in-place (SIP), with increased fentanyl and methamphetamine use. PWH successfully accessing substance use treatment services decreased post-SIP, highlighting the need for integrated substance use and HIV treatment.

With rising opioid overdose deaths nationally, there is concern that the coronavirus disease 2019 (COVID-19) pandemic may have disproportionately impacted vulnerable populations who experience syndemic conditions [1]—including people with human immunodeficiency virus (PWH) who experience co-occurring substance use/substance use disorders (SUDs) [2]. In the general population, 13% of Americans reported increasing or initiating substance use during the pandemic to cope with stress [3], and 16% of people who used drugs before COVID-19 reported increased drug use [4]. A scoping review estimated the rate of substance use in the general population during COVID-19 to be between 3.6% and 17.5% [5]. Moreover, an 18% increase in opioid overdoses has been documented nationwide [6]. PWH with SUDs disproportionately experience mental health conditions including depression, that may have been exacerbated by the pandemic and the public health response to COVID-19, including shelter-in-place (SIP) mandates, anxieties about COVID-19 infection, and decreased access to substance use and mental health services [7]. These impacts could affect populations of PWH disproportionately, including racial and gender minorities, given intersecting stigma, stress, and trauma among these groups [1, 8]. For instance, prior analyses documented disproportionately worsening human immunodeficiency virus (HIV) outcomes among Black PWH during COVID-19 [9] and a high prevalence of mental health and substance use concerns among transgender women with HIV during the pandemic [10].

It is important to note that worse outcomes along the HIV care continuum are consistently observed among PWH who use drugs compared to PWH who do not [11, 12]. Greater destabilization in viral suppression trajectories among people who inject drugs post–COVID-19 was previously shown in this cohort, highlighting the clinical importance of SUD on HIV outcomes [9]. However, despite the mounting evidence that substance use has worsened among the general population and particularly among underserved communities in the United States (US), few studies have examined how the pandemic has impacted the prevalence and predictors of SUD among PWH.

We therefore evaluated the impact of the COVID-19 pandemic on the prevalence of moderate or greater SUD risk among PWH enrolled in US HIV routine care, changes in the use of specific substances over time, and predictors of those who increased their SUD risk during the post–COVID-19 era. Understanding how the co-occurring epidemics of COVID-19 and HIV interact with the ongoing substance use epidemic in the US may help inform the development of multilevel intervention strategies both to improve HIV outcomes and reduce at-risk substance use and mortality.

## METHODS

### Study Population and Data Collection

This study was conducted within the Centers for AIDS Research Network of Integrated Clinical Systems (CNICS) cohort [13]. CNICS is a clinical cohort of PWH aged 18 and older in care at 10 sites across the US; 8 sites with relevant data were included in this study: Case Western Reserve University, Fenway Clinic, Johns Hopkins University, University of Alabama, University of California, San Diego, University of California, San Francisco, University of North Carolina, and University of Washington. CNICS harmonizes clinical data from the electronic medical record and assessment of patient-reported outcomes and measures (PROs). The PROs are tablet-based questionnaires of validated instruments that PWH self-report at the beginning of routine clinic visits [14]. The study cohort included PWH enrolled in CNICS who completed at least 1 CNICS clinical assessment of PROs during a routine visit in the pre/post-SIP time window between 1 March 2018 and 30 September 2022.

### Patient Consent Statement

All CNICS sites have local institutional review board approval to participate in CNICS, and de-identified data were used in analyses. Patients' written consent was obtained for study participation.

### Measures

Using the National Institute on Drug Abuse–Modified Alcohol, Smoking, and Substance Involvement Screening Test (NM-ASSIST) V2.0 [15], individuals were asked about their use of the following substances ever and in the past 3 months: cannabis, cocaine/crack, methamphetamines, inhalants, sedatives, or opioids obtained with or without a prescription. The ASSIST was originally developed by the World Health Organization and has demonstrated reliability and validity across research settings [16]. Following ASSIST scoring, for each drug reported, we created a Single Substance Involvement Score (SSIS). An SSIS between 0 and 3 indicates low risk, a score between 4 and 27 indicates moderate risk, and a score >27 indicates high risk [16]. Using these SSIS scores, we created a binary variable for moderate or high SUD risk, which was defined as having an SSIS >4 (yes/no).

Depression was assessed using the Patient Health Questionnaire-9 (PHQ-9), with moderate or high depression defined as a score >9 on the PHQ-9 [17]. We collected data on reported substance use treatment utilization during each PRO and created binary variables for any substance treatment received pre-SIP and post-SIP. Demographic variables including age in years, gender (male/female/transgender female; transgender men and gender not reported omitted from analysis due to small sample size), race/ethnicity (White/Black or African American/other racial category not previously listed, including Asian and American Indian), and geographic region (Northeast/South [included Case Western Reserve University]/West) were collected. Viral suppression was defined as a viral load <200 copies/mL (yes/no). A binary variable for SIP was created using the approximate average date that SIP was mandated across the study sites (19 March 2020) as the reference category.

### Statistical Analysis

**Figure 1.**
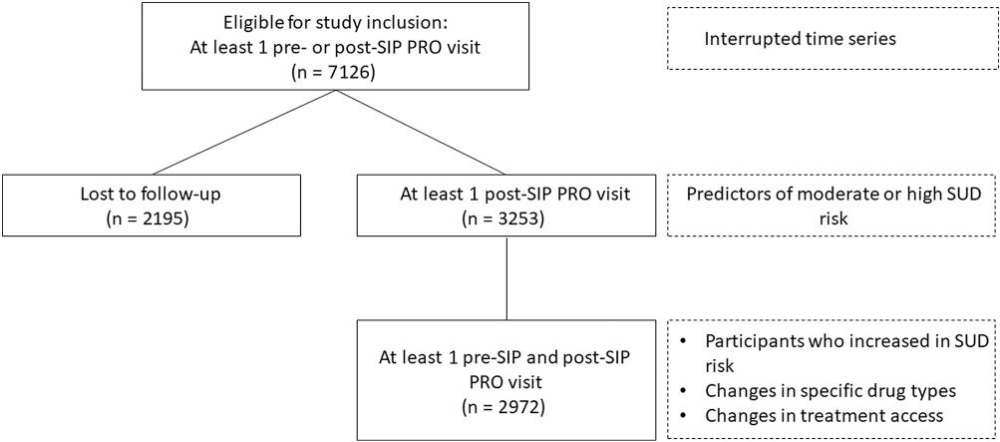
Consolidated Standards of Reporting Trials (CONSORT) diagram of participants included in each analysis. Subset of participants included in each analysis is presented in solid-line boxes, and specific analyses that used that subset of participants are presented in dashed-line boxes. Abbreviations: PRO, patient-reported outcomes and measures; SIP, shelter-in-place; SUD, substance use disorder.

Overall, the present study examined whether moderate/high SUD risk increased following the onset of the COVID-19 pandemic, identified the predictors of moderate/high SUD risk post-SIP, examined demographic and health-related differences between participants who increased from low-risk to moderate or high SUD risk compared to those who showed a decrease or no change in their SUD risk post-SIP, and estimated changes in the use of specific drug types and changes in the utilization of substance use treatment post-SIP compared to pre-SIP. We included all available data in each analysis, and sample sizes for each model are presented Figure 1. There were minimal missing data in predictors (<10% for all predictors), and as missingness was confined to the SUD outcome, maximum likelihood estimation was used to account for those who did not return for a substance use assessment, in a manner similar to multiple imputation [18].

First, an interrupted time series analysis (ITS) was used to evaluate whether there was a change in the slope of any moderate/high SUD risk over time, examining trends 2 years before and 2 years after SIP. ITS is a quasi-experimental design that estimates the impact of natural experiments, including public health policies, that have been implemented at a specific point in time on outcomes of interest (eg, substance use), by comparing the slopes preceding and proceeding the implementation of the policy [19]. Here, ITS treats SIP as an interruption in slopes to examine how SIP/COVID-19 altered SUD patterns. Analyses adjusted for age, race/ethnicity, gender, geographic location, and time-varying viral suppression. ITS analyses were conducted in the statistical software Stata [20] and all other analyses were conducted in R [21] using the packages lme4 [22], lmerTest [23], and emmeans [24] for mixed-effects models.

Second, we examined the predictors of moderate or high SUD risk post-SIP. This analysis was conducted using a logistic mixed-effects model, in which the outcome was the presence of moderate/high SUD risk over multiple observations per participant. Finally, we examined demographic (eg, age, race) and health-related differences (eg, viral suppression) between participants who increased in their SUD risk post-SIP compared to those who showed a decrease or no change in their SUD risk post-SIP using logistic regression.

Next, using a logistic mixed-effects model, we examined both person-level predictors (eg, age, gender) and time-varying predictors (eg, month, none/mild depression vs moderate/high depression, suppressed vs unsuppressed viral load) of moderate/high SUD reported during the post-SIP time period. We were primarily interested in examining compounding factors of risk—specifically, how worsening mental health outcomes among racial and gender minorities may interact to predispose participants to be at greater risk of moderate/high SUD [9, 10]. Therefore, we examined 3-way interactions between time, depression symptoms and gender, and time, depression symptoms and race, to examine how substance use may be modulated by depression symptoms over time differently as a function of gender or racial/ethnic identity. A random intercept of participant was included to account for repeated observations over time, and geographic region was included as an offset.

Then, we examined changes in the use of specific drug types pre/post-SIP. We conducted paired samples *t* tests to examine differences in the mean number of clinic visits in which participants reported using a specific drug within the past 3 months. Finally, we explored whether the reported utilization of substance use treatment decreased pre/post-SIP using a paired-sample *t* test.

## RESULTS

There were 7126 participants included in this study over 21 741 SUD assessments. The median age at first PRO assessment was 51 years (interquartile range [IQR], 39–58 years); 12% identified as Hispanic or Latino/Latina; 46% identified as Black/African American, 46% identified as White, 6% identified as another racial category (eg, Asian, Pacific Islander, American Indian), and 2% did not report racial/ethnic identity. There were 5532 cisgender men, 1449 cisgender women, and 95 transgender women (Table 1).

**Table 1.** Demographic and Clinical Characteristics of People With Human Immunodeficiency Virus in Clinical Care at 8 Sites Across the United States, March 2018–September 2022 (N = 7126)

Characteristic	No. (%)
Age, y, mean (SD)	48.92 (12.22)
Gender	
Male (cisgender)	5532 (78)
Male (transgender)	4 (0)
Female (cisgender)	1449 (20)
Female (transgender)	95 (1)
Not reported	46 (1)
Ethnicity	
Hispanic or Latino/Latina	853 (12)
Not Hispanic or Latino/Latina	5718 (80)
Not reported	555 (8)
Race	
White	3305 (46)
Black	3291 (46)
Other racial identity (eg, Asian, American Indian)	401 (6)
Not reported	129 (2)
Geographic location	
South	3329 (47)
Northeast	1628 (23)
West	2169 (30)
Viral suppression	
Suppressed at all visits	5764 (81)
Unsuppressed at any visit	1362 (19)
Depression	
None/mild at all visits	5336 (75)
Moderate/severe at any visit	1790 (25)
SUD	
None/mild at all visits	3612 (51)
Moderate/severe at any visit	3514 (49)
Treatment for SUD	
Never received treatment	6429 (90)
Received treatment at any visit	697 (10)
Time on ART, y, median (IQR)	9.5 (5–17)

Data are presented as No. (%) unless otherwise indicated.

Abbreviations: ART, antiretroviral therapy; IQR, interquartile range; SD, standard deviation; SUD, substance use disorder.

**Figure 2.**
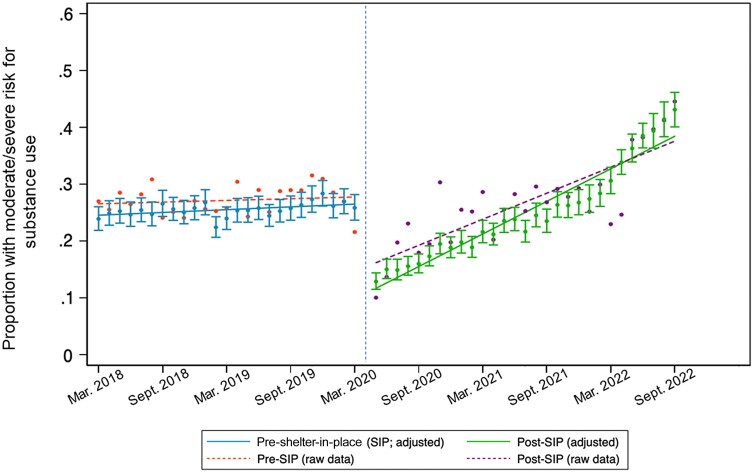
Substance use trends before and after coronavirus disease 2019 shelter-in-place (SIP) orders beginning in March 2020. Solid slope lines reflect the estimate in slope adjusted for age, race/ethnicity, gender, geographic location, and viral suppression; dashed slope lines reflect the unadjusted raw data.

In the ITS analysis, the rate of moderate/high SUD risk increased markedly during the pandemic after an initial decrease (Figure 2), with 24% having at least moderate SUD risk in March 2018, increasing to 43% at the end of analysis in September 2022. The difference in slope before and after the SIP order was statistically significant (adjusted odds ratio [AOR], 1.05%/month [95% confidence interval {CI}, 1.01%–1.09%/month]; *P* = .010).

**Figure 3.**
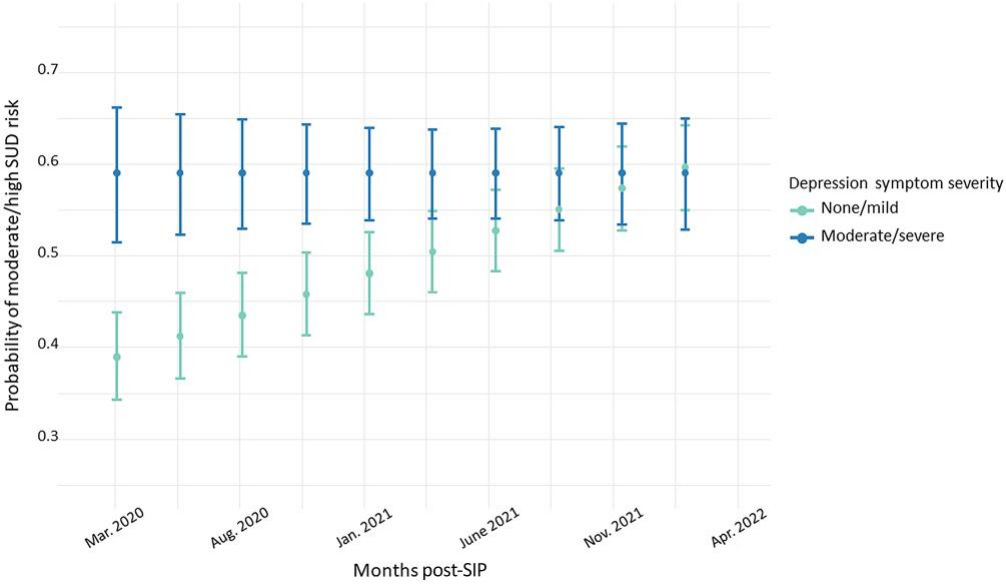
Participants with none/mild depression increased in the probability of moderate/high substance use disorder (SUD), and participants with moderate or greater depression demonstrated a consistently high likelihood of moderate/high SUD. Bars represent standard errors. Abbreviations: SIP, shelter-in-place; SUD, substance use disorder.

In a logistic mixed-effects model, we examined associations of moderate or high SUD risk across repeated observations for participants during the COVID-19 era. This model revealed significant main effects of age (per 10 years: AOR, 0.58 [95% CI, .54–.64]; *P* < .001) and viral nonsuppression (AOR, 1.79 [95% CI, 1.31–2.45]; *P* < .001) in which participants who were younger and virally unsuppressed at that measurement occasion were more likely to report moderate/high SUD risk. This model also revealed a significant 2-way interaction between time and depression symptoms: Those with none/mild depressive symptoms had markedly worsening SUD risk increased over time (AOR, 1.31%/month [95% CI, 1.01%–1.69%]; *P* = .041; Figure 3). In contrast, participants with moderate/severe depressive symptoms showed consistently high risk of substance use over time in the post–COVID-19 era.

**Figure 4.**
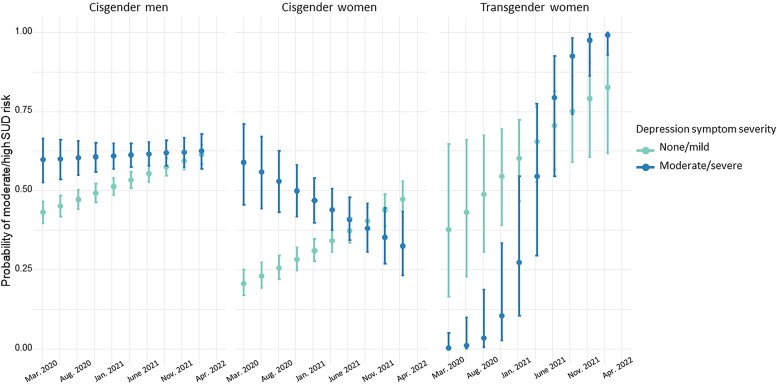
Illustration of the significant 3-way interaction between time (months since March 2020), depression (none/mild vs moderate/high depression), and gender, illustrating that the relation between depression and substance use disorder (SUD) risk over time differed as a function of gender identity. Error bars represent standard error.

In addition, there was a significant 3-way interaction between time, depression, and gender, which we further examined through pairwise comparisons of slopes across gender categories (Figure 4; *P* = .032). In examining SUD risk across gender categories, comparisons revealed lower odds of moderate/high SUD risk among cisgender women (AOR, 0.26 [95% CI, .08–.81]; *P* = .021) relative to transgender women. This comparison also revealed that transgender women with moderate or severe depression symptoms showed the greatest increase in moderate/high SUD risk over time, relative to cisgender women and men (Figure 4). There was no evidence of interaction between race/ethnicity, depression symptoms, and time for increased risk of SUD (*P* = .467).

**Figure 5.**
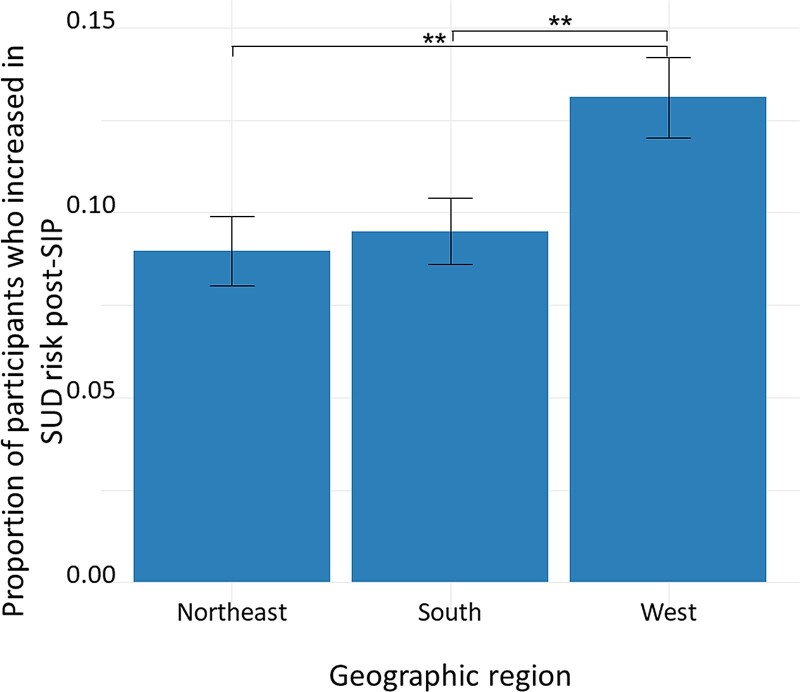
Proportion of participants in each geographic region who increased from none/mild substance use disorder (SUD) risk pre–shelter-in-place (SIP) to moderate/high SUD risk post-SIP. Error bars represent standard error. **P < .01.

We next examined predictors of increasing from no/mild SUD risk to moderate/high SUD risk (n = 312) compared to participants with no change or a decrease in risk (n = 2660). In a logistic regression, the likelihood of increasing to moderate/high SUD risk was predicted by geographic region, in which participants from the Northeast (AOR, 0.96 [95% CI, .93–.99]; *P* = .018) and South (AOR, 0.96 [95% CI, .94–.99]; *P* = .013) were less likely to increase in SUD risk relative to the West (Figure 5). There were no significant differences between the 2 other regions (*P* = .997) and no significant group differences in odds of increasing in SUD risk based on age, gender, racial/ethnic category, average pre-SIP PHQ-9 score, or pre-SIP viral suppression (ever unsuppressed) (all *P* > .100).

**Figure 6.**
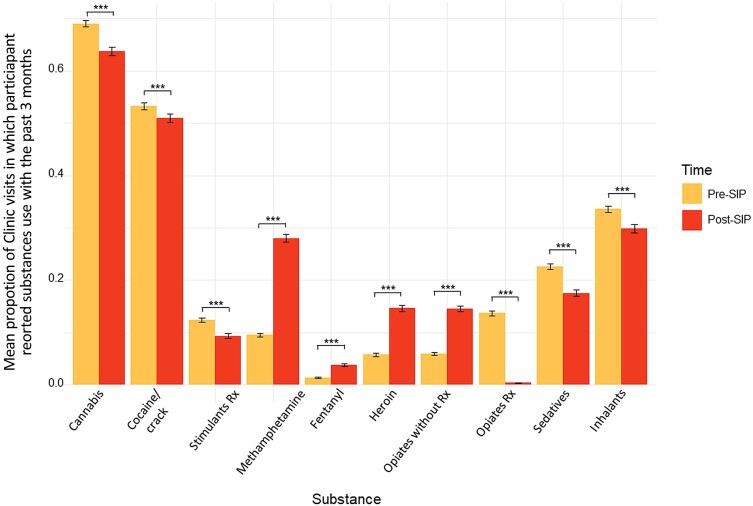
Mean proportion of patient-reported outcomes and measures in which participants reported using each drug within the past 3 months. Averaged over all pre–shelter-in-place (SIP) visits (shown in yellow) and post-SIP visits (shown in red). Error bars represent standard error. All comparisons were statistically significant, ***P < .001. Abbreviations: Cocaine, cocaine/crack; Rx, prescription; SIP, shelter-in-place.

Differences in the types of drugs used during the pre–COVID-19 era compared to the post–COVID-19 era were statistically significant for all substances assessed (all *P* < .001). Methamphetamine, heroin, and opioids obtained without a prescription showed the greatest reported increase in use during the COVID-19 era, whereas prescription opioids and sedatives showed the greatest reported decrease in use (Figure 6). Despite increases in substance use risk, participants were less likely to report receiving substance use treatment post-SIP compared to pre-SIP: *t*(2971) = 2.24 (*P* = .025).

## DISCUSSION

This study examined substance use before and after COVID-19 among PWH enrolled in a large multisite clinic-based cohort in the US and identified a near doubling in moderate or higher SUD risk during this time, increasing to 43% of the sample by study end. Substance use risk severity continued to increase >2 years following COVID-19. This rate of moderate or severe substance use is between 2.5 and 12 times higher than that reported in the general population during COVID-19 [5], and higher than drug use estimates in a large sample of participants diagnosed or at-risk of SUD during COVID-19 [25], suggesting that PWH were uniquely impacted by COVID-19. The continued dramatic increase in at-risk substance use over time highlights the urgent need for systems for monitoring and addressing substance use, and for substance use treatment services to be integrated within HIV primary care.

Those who were younger and virally unsuppressed were more likely to newly report moderate/high SUD risk during the post-SIP era. Younger individuals may have been disproportionately impacted by the pandemic's socioeconomic impacts due to being at earlier stages of their careers, having fewer resources, less resilience to impacts of social isolation, and/or other factors [19]. While younger individuals were more likely to report moderate/high substance use risk post–COVID-19, substantial substance use risk was distributed across the lifespan among this sample of PWH [26]. Both moderate/high SUD risk and unsuppressed viral load post–COVID-19, which worsened among most groups post–COVID-19 [9], are likely due to upstream social determinants of health that impact both SUD risk and antiretroviral therapy adherence (eg, housing security, stigma, decreased care engagement for both substance use treatment and HIV care [27]). Alternatively, worsening substance use after COVID-19 could lead to worsening virologic suppression. In either case, programs that integrate HIV care with substance use treatment are likely to have the greatest impact (ie, co-provision of long-acting antiretroviral therapy with cabotegravir/rilpivirine and substance use treatment with long-acting buprenorphine for opioid use or naltrexone for methamphetamine use).

This model also revealed a significant interaction between depression symptoms and time post-SIP: those with no/mild depression symptoms showed greater increases in the likelihood of reporting moderate/high SUD risk post-SIP. By the end of the time period we measured, those with no/mild depression symptoms reported similar substance use risk to those with depression symptoms of greater severity. It is unclear why those with milder depression symptoms had rising substance use risk following COVID-19, while those with more severe depression symptoms maintained a high prevalence of higher-risk substance use over time. This may be because other mental health symptoms contributed more significantly to SUD risk, such as anxiety or loneliness/social isolation [28]. Alternatively, other factors may contribute to the rising substance use post–COVID-19 that disproportionately impacted those who previously had less substance use involvement, including increasing popularity of methamphetamine among sexual minority men across the US, including as part of chemsex [29], greater availability of fentanyl at a time of decreasing access to prescription opioids [30, 31], and/or socioeconomic effects of the pandemic leading to less access to employment and non–substance use–related leisure activities [32]. While cisgender women and men with more severe depression symptoms did not experience worsening SUD risk post-COVID-19, transgender women with depression symptoms had markedly worsening SUD risk post-COVID-19. It is possible that transgender women, a population already impacted by intersectional stigma, discrimination, social isolation, and high rates of mental health comorbidity at a time of decreased access to mental health and substance use treatment services, may have experienced disproportionate worsening of substance use over time [10]. Cisgender women living with HIV had lower SUD risk during COVID-19, which is similar to what has been seen in other cohorts before and after COVID-19 [33, 34]. There were also differences in SUD risk by geographic region, in which participants from the West were more likely to increase from none/mild SUD risk pre–COVID-19 to moderate/severe SUD risk during the COVID-19 era, aligned with research suggesting an increase in fentanyl on the West Coast that has lagged a few years behind the East Coast, as well as a worsening methamphetamine epidemic [35]. Differences SIP restrictions across the US may also have influenced substance use risk, given increases in accidental overdose deaths [36] and overdose emergency department visits [37] after SIP orders, potentially due to increased social isolation in regions with stricter restrictions.

Our study also found a significant increase in the use of heroin, methamphetamine, and opioids obtained without a prescription following COVID-19, and significant decreases in the use of prescription opioids and sedatives. Decreased access to medical providers during COVID-19 may have led participants to seek substances that were more widely available outside of the medical system. Although methadone clinics generally increased follow-up windows with increased take-home doses during this period, this was likely insufficient to counter overall trends in substance use severity at a time of decreased in-person service availability, with less patient contact and less access to other services such as counseling, buprenorphine, naltrexone, and methamphetamine treatment programs. This is unfortunate in the context of increasing opioid overdose deaths nationwide, in part attributed to increased access to fentanyl throughout the US [2, 38]. Reported increases in polysubstance use, including with methamphetamine, potentially contributes to opioid overdose risk [2, 38]. Finally, participants were significantly less likely to report engaging in substance use treatment during the post–COVID-19 era compared to the pre–COVID-19 era, despite increases in moderate/high SUD risk, likely due to decreased access to these services, potentially contributing to worsening substance use severity over time [39].

These findings should be interpreted within the context of certain limitations. Data for this study were collected among a cohort of PWH enrolled in HIV care; therefore, these findings may not be generalizable to those not engaged in care. While we sought to account for potential confounding, it is possible that unmeasured confounding affected our results. Participants who were lost to follow-up may have been the most vulnerable participants who did not reengage in care. Thus, the increase in higher-risk SUD presented may be an underestimate of the true effect. In addition, it is possible that participants at greatest risk of moderate/high SUD were slower to reengage in care post-SIP, as reflected in an initial decrease in SUD risk in our ITS analysis. We sought to address missingness using maximum likelihood estimation, which has been found to be both reliable and efficient as compared to multiple imputation [18]. Additionally, future research should further examine both depression and SUD risk among a larger sample of transgender women, given that this group showed a steep upward trend and trajectory distinct from cisgender women and men, although the sample for this group was smaller than other populations. Further, our findings examining changes in which substances were used before and after onset of the COVID-19 pandemic may understate the increase in fentanyl use in this sample given that participants are often unknowingly using fentanyl with other substances [38].

In summary, we found a significant increase in moderate/high SUD risk among PWH in a large multisite network of HIV clinics throughout the US from 2018 to 2022. This rising prevalence could be related to an increase in social isolation and mental health symptoms, as well as reduced access to substance use and HIV treatment caused by disruptions due to the pandemic, which have persisted years after pandemic onset in some jurisdictions due to ongoing challenges with funding and staffing [40]. To address the combined epidemics of substance use and HIV following the COVID pandemic, a renewed investment in integrated substance use and HIV treatment is vital.

## Notes

***Financial support*.** This work was funded by the National Institute of Allergy and Infectious Diseases (grant numbers R01AI158013 to M. A. S., M. G., and M. O. J.; R24AI067039 to K. A. C. and M. O. J.; P30AI027763 to M. G.; and K24 AI167805 to K. A. C.); the National Institute of Mental Health (grant number P30MH062246 to M. O. J.); and the National Institute on Drug Abuse (grant numbers U01DA036935 to R. D. M., R01DA047045 to H. M. C., and 1K01DA056306 to J. P. J.), all of the National Institutes of Health (NIH).
